# Robotic Surgery for the Treatment of Achalasia Cardia: Surgical Technique, Initial Experiences and Literature Review

**DOI:** 10.7759/cureus.21510

**Published:** 2022-01-23

**Authors:** Mustafa Uzunoglu, Fatih Altintoprak, Omer Yalkin, Kayhan Özdemir

**Affiliations:** 1 General Surgery, Bursa City Hospital, Bursa, TUR; 2 General Surgery, Sakarya University, Serdivan, TUR; 3 Surgical Oncology, Bursa City Hospital, Bursa, TUR

**Keywords:** treatment, robotic surgery, heller myotomy, esophagus, achalasia

## Abstract

Background

The outcomes of surgical interventions for achalasia treatment improved with the advent of minimally invasive surgery and the introduction of robotic surgery. This article describes the technical details of robotic achalasia surgery, shares our initial experiences, and discusses why robotic surgery will become the first choice for the surgical treatment of achalasia.

Methods

The records of patients with a diagnosis of achalasia who underwent robotic surgery were evaluated retrospectively. The patients’ data were examined in terms of demographic parameters, duration of complaints, treatment options applied previously, robotic surgery technique, and postoperative outcomes.

Results

Of the six patients evaluated, four (66.7%) were males and two (33.3%) were females. Their mean age was 32 years (20-51 years), and the mean symptom duration was 4.6 years (2-9 years). All of the patients underwent robotic Heller cardiomyotomy surgery. After the myotomy procedure, five of the six patients (83.3%) underwent partial anterior fundoplication (Dor) as an antireflux procedure. The cruroraphy procedure was performed in one patient (16.7%) due to accompanying hiatal hernia, whereas the procedures were completed in five patients (83.3%) without performing posterior dissection of the oesophagus. In the postoperative follow-up period, no surgical problem was encountered, while reflux symptoms developed in one patient (16.7%) and were controlled by medical therapy.

Conclusions

The success of surgical treatment of achalasia is incontrovertible. Due to the various advantages of robotic surgery, it is now frequently used in narrow-area surgeries, such as achalasia surgery.

## Introduction

Achalasia cardia is a neurodegenerative motility disorder involving impaired oesophageal peristalsis and loss of lower oesophageal sphincter function [1]. Current treatment modalities for achalasia cannot prevent or reverse the neurodegeneration [2]. Therefore, treatment options for patients with achalasia have two goals, namely reducing (various medical treatments and/or botulinum toxin injection) or destroying (endoscopic balloon dilatation and/or surgical treatment) the tonus of the lower oesophageal sphincter.

The option of destroying the lower oesophageal sphincter can be traced to the whale-bone dilation experiments of Sir Thomas Willis in the 1600s [3]. The historical development continued with the application of the first balloon dilatation in 1887, description of surgical myotomy by Ernest Heller, incorporation of antireflux procedures in myotomy, introduction of minimally invasive surgery, per-oral endoscopic myotomy (POEM), and current robotic surgical procedures [3-6].

Robotic surgery will likely become the preferred method for achalasia surgery due to its advantages over classical minimally invasive methods. We describe here the technical details of robotic achalasia surgery and discuss why robotic surgery will become the first choice for the surgical treatment of achalasia.

## Materials and methods

The records of patients with achalasia who underwent robotic surgery with da Vinci S Robotic System (Intuitive Surgical, Sunnyvale, CA, USA) in Sakarya University General Surgery Department from June 2016 to November 2017 were evaluated retrospectively. Permission for this study was obtained from Sakarya University Faculty of Medicine Clinical Research Ethics Committee (02/02/2019-E.14983). The patients’ data were examined in terms of demographic parameters, duration of complaints, previous treatments, robotic surgery technique, and early postoperative outcomes.

No financial support that could have adversely affected the decisions made in the study was received from any company that had a direct link to the research subject, companies, or any commercial entity that supplied and/or produced medical instruments, equipment, or materials, or political entities.

Preoperative assessment

To confirm the diagnosis and exclude pseudo-achalasia in patients with clinical complaints consistent with achalasia, upper gastrointestinal radiography with barium swallow, upper gastrointestinal system endoscopic examination, thoracoabdominal computerised tomography, and oesophagus manometric examination are performed. Following confirmation of the achalasia diagnosis, the patients are prepared for surgery.

Patient positioning and port placement

**Figure 1:**
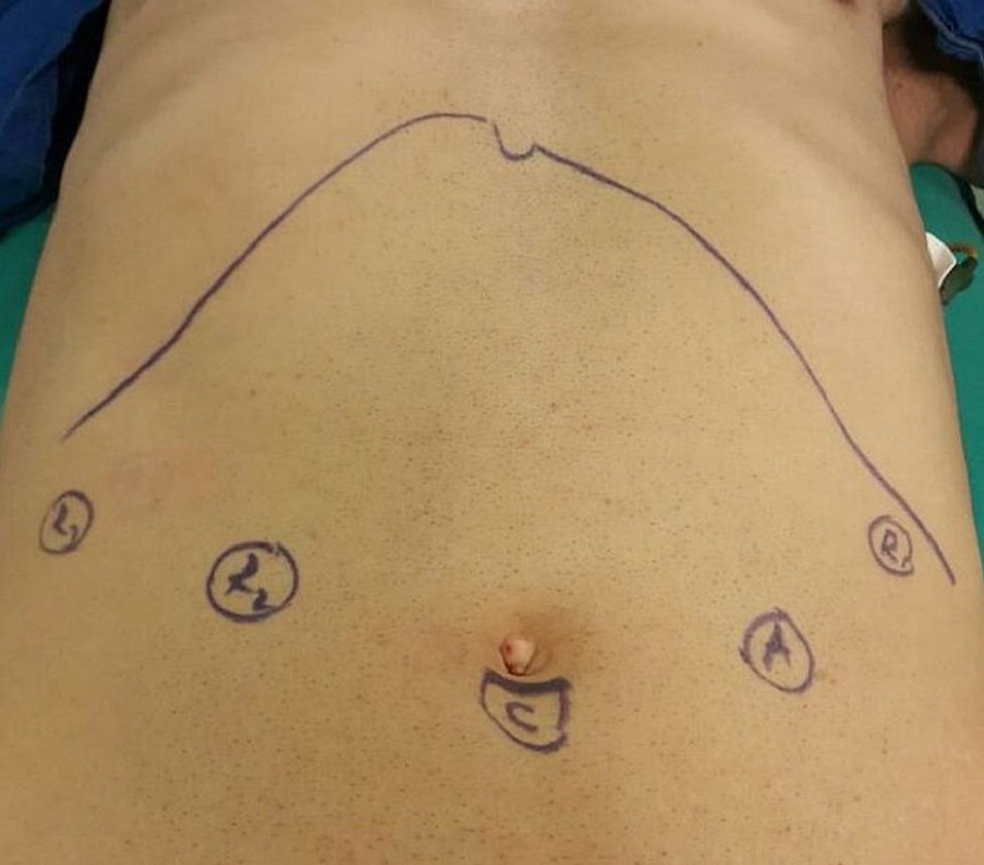
Port localizations (C, camera; R1, robotic first arm; R2, robotic second arm; R3, robotic third arm; A, assistant port)

Following endotracheal intubation and nasogastric tube placement, the patient is placed in the supine position with the lower extremities and right upper extremity covered and the left upper extremity open. Abdominal wall cleaning and sterile covering are completed. After marking the insertion sites of the ports using a sterile pen (Figure 1), CO2 insufflation is initiated using a Veress needle inserted under the subumbilical port localization and pneumoperitoneum until an intraabdominal pressure of 12 mm/Hg was reached. After whole intraabdominal visual exploration, other operative ports are placed under direct vision. Ports localizations are shown in Figure 1.

The operating table is positioned so that the head of the patient is elevated 30°. The da Vinci S robotic system (Intuitive Surgical, Sunnyvale, CA, USA) is approximated from the head side of the patient and the docking procedure is completed. Next, the operation is initiated after inserting the camera and operative tools through the ports (subumbilical port, camera; port 1, harmonic scalpel; port 2, bipolar forceps; port 3; Cadiere forceps; assistant port, atraumatic grasper).

Surgical technique

Freeing of Fundus and Oesophagus

**Figure 2:**
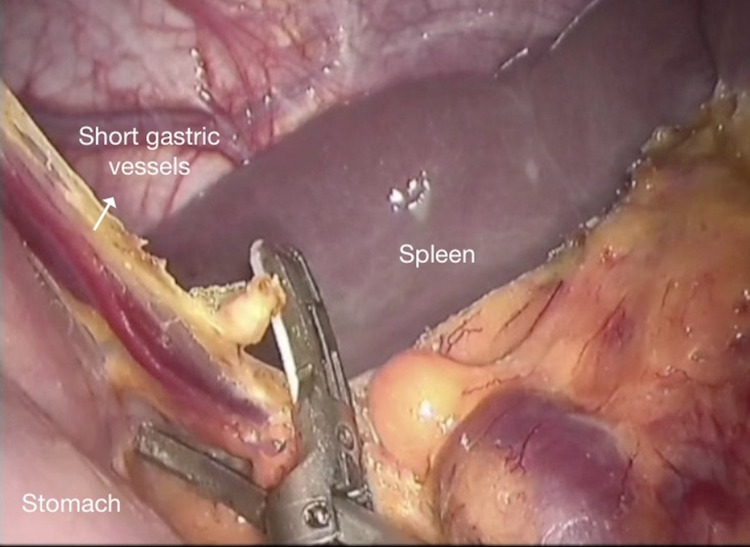
Freeing of the gastric fundus using a harmonic scalpel

Using the Cadiere forceps inserted through port 3, visual exploration of the oesophagogastric region is completed by retracting the left lobe of the liver. Dissection of the omentum majus from the stomach at the proximal one-third level of the greater curvature is initiated. Using the harmonic scalpel, dissection is continued proximally and the gastric fundus is freed up to the left hiatal crus, including the short gastric vessels (Figure 2).

**Figure 3:**
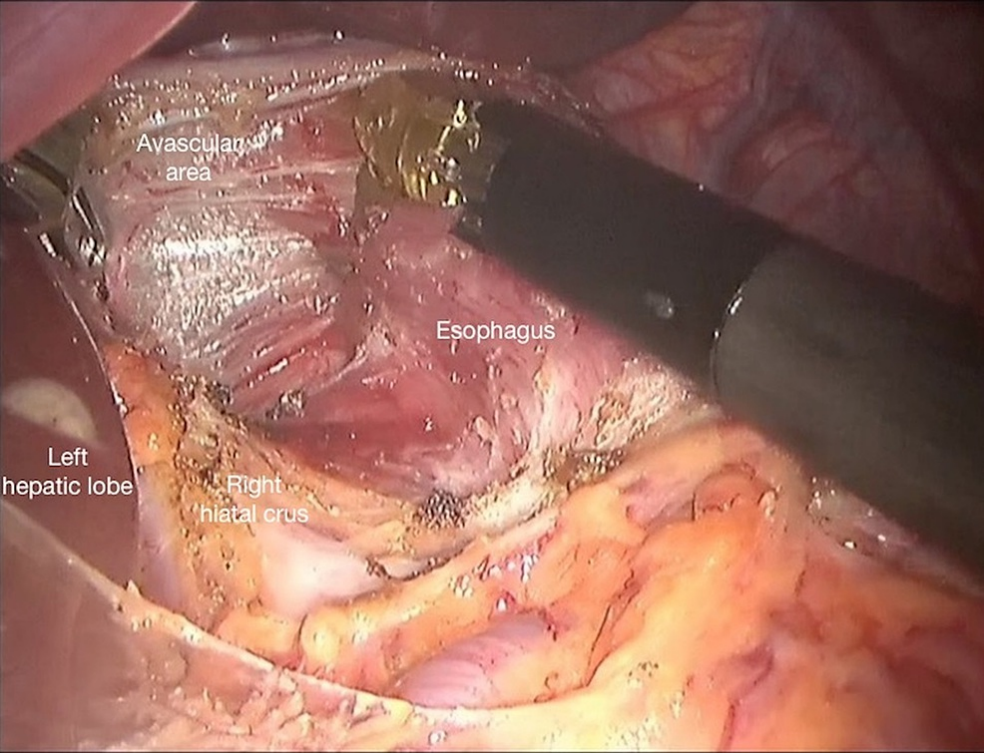
Freeing of the oesophagus through the avascular plane cranially

Dissection is initiated on the lesser curvature side of the stomach and the omentum minus is opened using the harmonic scalpel, preserving the hepatic branch of the vagus nerve. The dissection is continued until the right diaphragmatic crus is exposed. The anterior freeing of the oesophagogastric region is completed by opening the phreno-oesophageal ligament and the fascia of Laimer. Starting from the level of the right diaphragmatic crus, the oesophagus is freed over the avascular planes towards the mediastinum. These procedures are repeated along the anterior and left lateral walls of the oesophagus, and freeing of the oesophagus 8-9 cm proximally is completed without entering the posterior plane (Figure 3).

Myotomy

**Figure 4:**
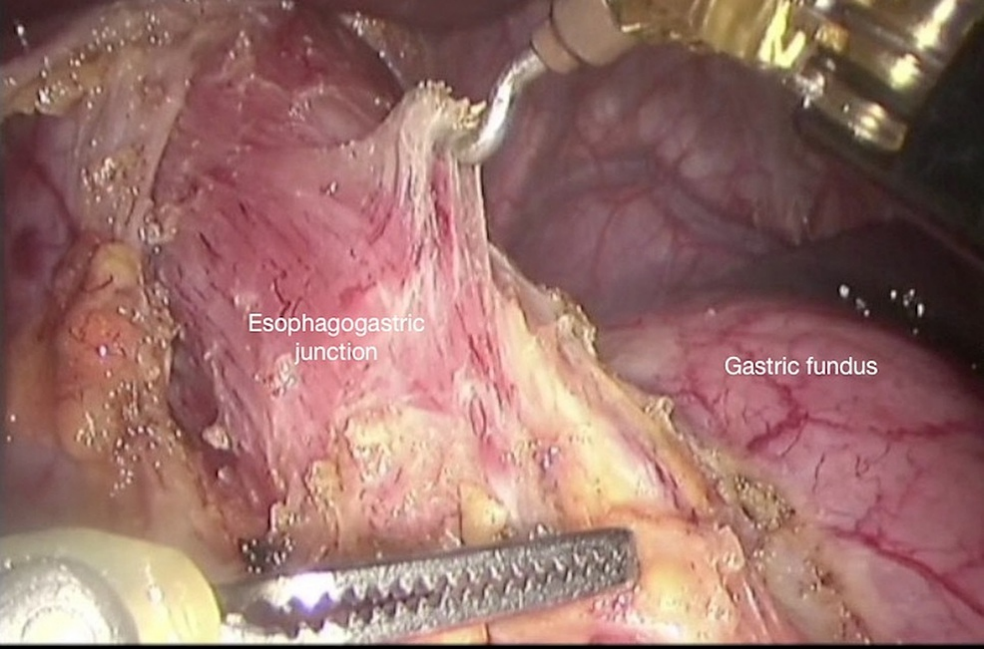
Initiation of myotomy by opening the longitudinal muscle fibres at the level of the oesophagogastric junction

The stomach is pulled distally using the atraumatic grasper inserted through the assistant port, and the line on which myotomy would be performed is determined. The fat pads located on the midline at the oesophagogastric junction and on the myotomy line towards the stomach are excised using the harmonic scalpel. The left vagus nerve over the anterior wall of the oesophagus is visualised and lateralised to the left side, and the myotomy procedure is started from the level of the oesophagogastric junction (Figure 4).

**Figure 5:**
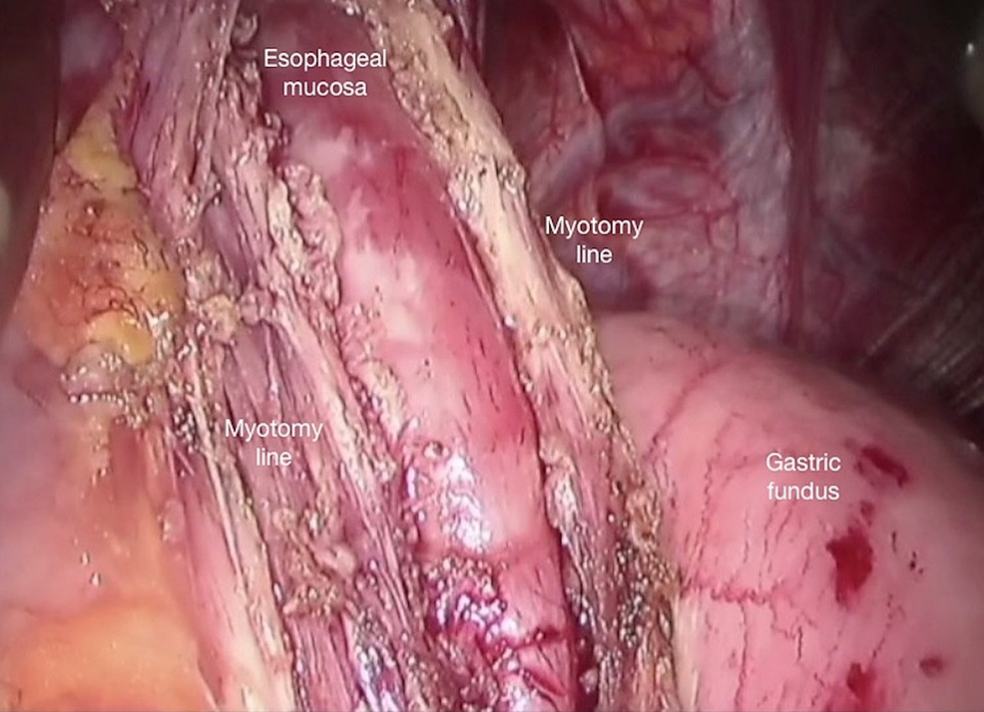
Completed proximal myotomy: the entire muscle layer on the oesophageal mucosa is dissected

The myotomy procedure is performed using a hook cautery inserted through port 1 via a sharp dissection on a low amperage (15-18 milliamperes). The longitudinal and circular muscle fibres of the oesophagus are cut to gain access to the submucosal area. The myotomy is continued proximally for 8-9 cm (Figure 5).

**Figure 6:**
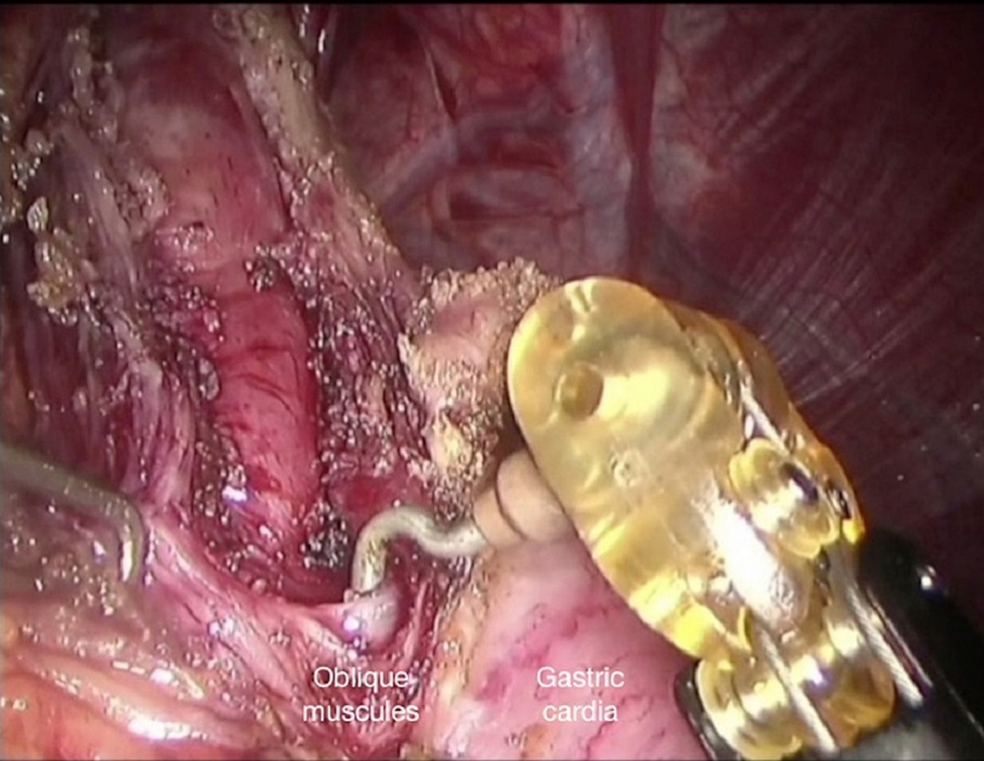
Completing the distal myotomy: dissecting the oblique muscles using a robotic hook

A distal myotomy procedure is initiated following dissection of all longitudinal and circular fibres and adequate mucosal freeing. The myotomy is completed in the submucosal area including the oblique muscles (Figure 6) so that the initial oesophagogastric junction would be at least 2 cm from the proximal stomach.

Control of Myotomy Line

After controlling the length of the myotomy line and adequacy of myotomy visually, a standard oesophagogastroduodenoscopic control procedure is initiated. After passing an endoscope through the distal oesophagus into the stomach without difficulty (by visually tracing the light after the light of the intraabdominal camera is closed), the surgical field is filled with saline and an air-leak test is performed using the endoscope. The fundoplication procedure is initiated after ensuring control of the myotomy line.

Fundoplication

**Figure 7:**
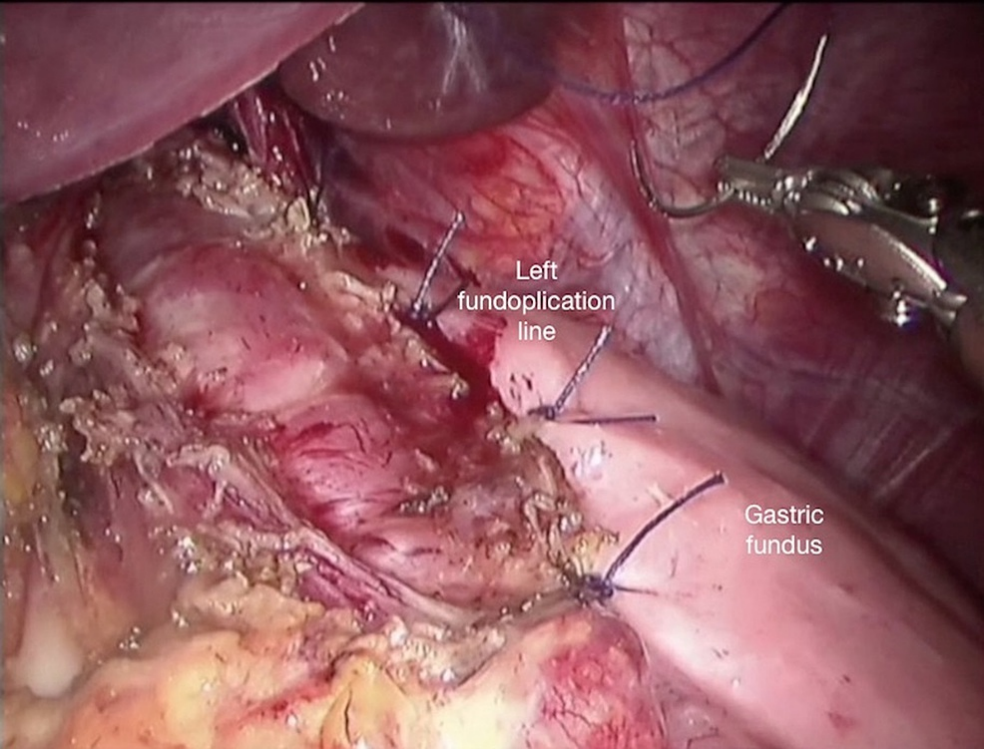
Completed left side of the fundoplication

A needle-driver is placed through ports 1 and 2 for partial anterior (Dor) fundoplication. Using 2/0 absorbable suture material, the fundus of the stomach which is close to the left myotomy line is secured to the left lateral myotomy line (Figure 7).

**Figure 8:**
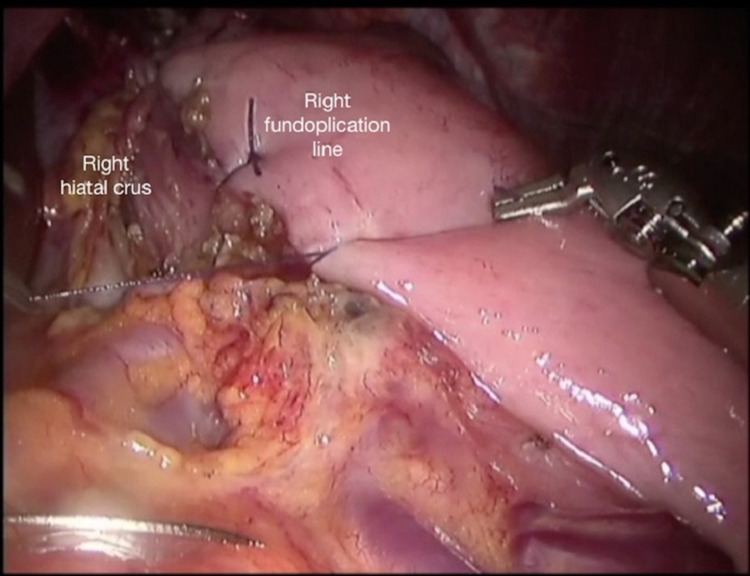
Final form of the partial anterior (Dor) fundoplication

This procedure is carried out using at least three sutures. The left crus is not secured. The fundus of the stomach, which is at least 3 cm lateral to the first suture line, is sutured to the right lateral myotomy line. Again it is sutured one by one and at least three sutures are placed (Figure 8).

To prevent rotation of the fundoplication and maintain its position, at least one of the sutures in the right myotomy line is made so that it would pass through the right crus.

Following completion of fundoplication, haemostasis control is performed, the nasogastric tube is removed, one aspirative drain is placed in the surgical field, and the procedure is terminated. After checking the looseness of the robotic arms, they are removed. Following the evacuation of intraabdominal air, the ports are removed. No fascia closure is performed at the port sites.

Postoperative assessment

Following extubation and recovery, the patients undergo clinical follow-up. An endoscopic examination is conducted using water-based contrast material in patients who are mobile on postoperative day 1. A liquid oral diet is initiated and the aspirative drain is removed. Endoscopic examination reveals that the oesophagogastric passage is normal and there is no contrast leakage.

## Results

Of the six patients, four (66.7%) were males and two (33.3%) were females. Their mean age was 32 years (20-51 years) (Table 1).

**Table 1:** Demographic features of the patients

Gender	n (%)	
Female	2	33.3
Male	4	66.7
	Mean	Min-Max
Age	32	20-51

The mean duration of their complaints was 4.6 years (2-9 years). One patient (16.7%) had a history of two unsuccessful endoscopic balloon dilatation procedures. All of the patients underwent robotic Heller cardiomyotomy surgery. After myotomy, five patients (83.3%) underwent partial anterior fundoplication (Dor) as an antireflux procedure; no antireflux procedure could be performed in the remaining patient (16.7%) (history of two unsuccessful balloon dilatations) due to diffuse adhesions between the gastric fundus and diaphragm. The procedure was performed in one patient (16.7%) due to accompanying hiatal hernia, whereas the procedures were completed in five patients (83.3%) without performing posterior dissection of the oesophagus. The mean operation duration was 165 min (range, 150-180 min) (mean console duration, 103 min; range, 95-110 min). No surgical problem was encountered during the early postoperative follow-up period, while a reflux complaint controlled by medical therapy developed in one patient (16.7%, the patient in whom fundoplication was not performed). The mean hospital stay was 3.6 days (2-9 days). No disease relapse was detected during the postoperative clinical follow-up (55 months; range, 48-60 months). Clinical data of the patients are given in Table 2.

**Table 2:** Clinical variables of the patients

	Mean	Min-Max
Complaint's duration (years)	4.6	2.9
Operation duration (min)	165	150-180
Console duration (min)	103	95-110
Hospital stay (days)	3.6	2.9
Follow-up duration (months)	55	48-60
	n (%)	
Partial anterior fundoplication		
Yes	5 (83.3%)	
No	1 (16.7%)	
Surgery-related complication		
Yes	1 (16.7%)	
No	5 (83.3%)	
Relapse during follow-up		
Yes	0 (0)	
No	100 (100)	

## Discussion

The use of robotic systems in achalasia surgery began in 2000; the results of studies of multiple cases were reported beginning in 2005. Horgan et al. [7] compared the early results of laparoscopic and robotic surgery in 121 patients; the most important differences between the two methods were the operation duration (mean, 141 min) and the rate of mucosal perforation. Although the operation duration was longer in the robotic surgery arm, the mucosal perforation rate was 16% in the laparoscopic group and 0% in the robotic surgery group. Another study published in the same year of 104 patients with achalasia reported that the operation duration was longer with robotic surgery but decreased with increasing operator experience (from 162 to 113 min). The rate of mucosal perforation was 0% [8]. Subsequently, the operation duration reached similar levels to laparoscopic surgery due to increased experience and developments in robotics. Sanchez et al. [9] reported that the operation durations of robotic and laparoscopic surgeries were similar, and that the mucosal perforation rate was 5.5% in the laparoscopic group and 0% in the robotic group.

The low risk of oesophageal mucosal perforation with robotic surgery is due to its superior technical features compared with laparoscopic surgery. Ballouhey et al. [10] stated that this difference was due to the three-dimensional image produced during robotic surgery, which also gives a sense of depth, and the use of articulating instruments. The use in myotomy of articulated tools (such as scissors and hooks) enables parallel entry to the submucosal field and dissection of overlying muscle fibres without applying pressure on the mucosa. This advantage is important for retrograde myotomy toward the stomach. The importance of articulating tools in achalasia surgery has been reported [7], as these facilitate repair of perforations. We agree that the above-mentioned technical advantages are important, particularly in distal myotomy toward the stomach. Moreover, these advantages explain the superiority of robotic myotomy to laparoscopic myotomy in terms of the mucosal perforation rate. Due to the presence of oblique muscle fibres, the submucosal area is narrow and is the most frequent site of mucosal perforations.

Multicenter retrospective studies have assessed the efficacy of robotic surgery for achalasia. A retrospective study of 2683 patients with achalasia (418 open, 2116 laparoscopic, 149 robotic) who underwent surgical treatment indicated that robotic surgery was superior to open surgery [11]. Although robotic surgery was superior in terms of mortality, morbidity, intensive care requirement, hospital stay duration, and readmission outcomes in the early period, the differences were not significant [11]. The marked difference in the number of patients who underwent robotic and laparoscopic surgery is notable.

The achalasia surgery subdivision of a systematic review and meta-analysis (31 trials, 2166 patients) of laparoscopic and robotic abdominal surgeries concluded that the operation duration and hospital stay duration were not different, that the risk of mucosal perforation in robotic surgery was eightfold lower than in laparoscopic surgery, and that the quality of life was better in the robotic surgery group [12].

The 2012 SAGES [13] achalasia surgical treatment guidelines state that laparoscopic surgery is superior to open surgery in all respects and the rate of intraoperative mucosal perforation was lower with robotic surgery: at the time, the mean intraoperative mucosal perforation rate using laparoscopic surgery was 6.9% (range, 0-33) and the mean intraoperative mucosal perforation rate using robotic surgery was 0.7% (range, 0-3).

Milone et al. [14] compared laparoscopic and robotic Heller myotomy and reported significant lower rate of intraoperative esophageal perforations (OR = 0.13, P < 0.001, 95% CI 0.04, 0.45) in robotic approach, respectively. Luo et al. reported robotic surgery was similar or superior to laparoscopic surgery in terms of intraoperative and postoperative complications in a review of 98 articles related to the suitability of robotic surgery for foregut surgery [15].

The utility of robotic surgery is limited due to its high cost. Shaligram et al. [11] reported that the cost of laparoscopic Heller myotomy was $7.441 ± 7.897 compared to $9.515 ± 5.515 for robotic surgery. Other studies including inguinal hernia repair [16] and distal pancreatectomy [17] have also reported that robotic surgery is more costly than laparoscopic surgery. We agree that cost accounting of robotic surgery is important despite its technical advantages. Reducing the cost of robotic surgery will lead to it becoming the first choice for diverse surgical procedures.

Recent studies showed that in the surgical treatment of achalasia, it is recommended to perform complete myotomy including all fibers and myotomy line should be long enough to include the proximal gastric area which is the crucial factor associated with a lower recurrence rate of achalasia. Also, lower post-operative Eckardt score was found in patients undergoing robotic myotomy, comparing laparoscopic approach. Robotic surgery makes this possible with very low complication rates to its technical capabilities [18, 19].

Peroral endoscopic myotomy (POEM) is an endoscopic method that has been used in the treatment of achalasia in recent years. In patients with achalasia, an endoscopic submucosal tunnel is created and myotomy is performed starting from the distal esophagus and extending to the cardia of stomach [18]. Postoperative Eckardt score was significantly lower, postoperative dysphagia remission was remarkably improved and more cost‑effective in POEM than in Heller myotomy cases. However, postoperative gastroesophageal reflux disease (GERD) rate was higher in POEM than surgical Heller myotomy groups [20-21].

## Conclusions

The findings of this study indicate that robotic surgery can be performed effectively in patients with achalasia. No surgery-related major complication was observed in the follow-up period. Only one patient developed reflux, which was controlled with medical therapy. Robotic surgery for achalasia has various technical advantages, which result in a lower rate of perioperative mucosal perforation, providing a lower recurrence rate and more complete myotomy leading to more reliable long-term results.
